# Association of Three Common Single Nucleotide Polymorphisms of ATP Binding Cassette G8 Gene with Gallstone Disease: A Meta-Analysis

**DOI:** 10.1371/journal.pone.0087200

**Published:** 2014-01-30

**Authors:** Zhao-Yan Jiang, Qu Cai, Er-Zhen Chen

**Affiliations:** 1 Department of Surgery, Shanghai Institute of Digestive Surgery, Shanghai, China; 2 Department of Emergency, Ruijin Hospital, Shanghai JiaoTong University School of Medicine, Shanghai, China; State Key Laboratory of Reproductive Biology, Institute of Zoology, Chinese Academy of Sciences, China

## Abstract

**Background:**

In this study, we evaluated the association between these polymorphisms and gallstone disease using meta-analysis and compared the hepatic ABCG5/G8 mRNA expression and biliary lipids composition in patients with different genotypes of T400K and Y54C.

**Methods:**

Data were analyzed using the Stata/SE 11.0 software and a random- effects model was applied irrespective of between-study heterogeneity. Hepatic mRNA expression of ABCG5/G8 genes in 182 patients with gallstone disease and 35 gallstone-free patients who underwent cholecystectomy were determined using real-time PCR. Genotypes of Y54C and T400K in the ABCG8 gene were determined by allelic discrimination using either genomic DNA or hepatic cDNA as template by Taqman assays. Biliary compostion in gallbladder bile was assayed in these patients as well.

**Results:**

Ten papers including 13 cohorts were included for the final analysis. In the genotype model, the overall association between genotype with gallstone was significant for D19H (OR = 2.43, 95%CI: 2.23–2.64, P<0.001), and for Y54C (OR = 1.36, 95%CI: 1.01–1.83, P = 0.044), or T400K (OR = 1.17, 95%CI: 0.96–1.43. P = 0.110). In allele model, minor alleles of D19H polymorphism (allele D: OR = 2.25, 95%CI: 2.10–2.42, P<0.001) and of T400K polymorphism (allele K: OR = 1.18, 95%CI: 1.06–1.31, P<0.001) were related with an increased risk of gallstone disease. However, minor allele of Y54C polymorphism (allele Y, OR = 1.08, 95%CI: 0.96–1.21, P = 0.146) was not related with gallstone disease. *I* ^2^ statistics indicated no significant between-study heterogeneity for all genetic models for any of the three polymorphisms. Funnel plot and Egger’s test suggested the absence of publication bias as well. However, no association of T400K and Y54C polymorphism with hepatic ABCG8/G5 mRNA expression or biliary lipids composition was found.

**Conclusions:**

Our study showed strong association of D19H polymorphism with gallstone disease. T400K and Y54C polymorphism, though to a less extent, may also relate with gallstone disease.

## Introduction

Cholesterol gallstone disease is common in western countries [1]. In Shanghai city, its prevalence is increasing to 10.8% in the population with an addition of 2.97% subjects have a previous cholecystectomy. The majority of the gallstones are of cholesterol type.

The classical pathogenesis model of cholesterol gallstone formation included three defects: supersaturation of biliary cholesterol, imbalance of pro/anti-nucleation factors and impaired gallbladder motility [2]. Recently, genetic factors, as the predisposition to gallstone disease and interacts with environmental factors, have drawn keen attention of researchers [3]. Katsika et al [4], showed that genetic heredity contribute 25% of factors to gallstone formation after an elegant analysis of data from Swedish twins. Since late 1980s’, studies have been attempting to reveal susceptible genes associated with gallstone disease in different populations. The possible genes studied include apolipoprotein E [5]–[8], B [7], [8], cholesterol 7alpha-hydroxylase [7], [8], etc. In the last decade, with the understanding of ATP binding cassette (ABC) G5 and G8 as major cholesterol transporters in hepatic and intestinal cholesterol secretion and in regulating biliary cholesterol content and cholesterol absorption [9], studies on association of polymorphism of ABCG8 and gallstone disease have been published [10]–[19]. The most studied loci are D19H, T400K and Y54C. Due to difference in allele frequency at each polymorphic locus between different ethnicities, the associations between these SNPs with gallstone disease are somewhat not consistent. Thus, a meta-analysis approach to evaluate the association between each loci and gallstone disease is worth being performed. Furthermore, the functional roles of these polymorphic loci are not fully clarified yet. No study has ever determined whether any difference exists for the hepatic expression of ABCG8 or ABCG5 genes between carriers of different alleles.

The aims of our study are: 1) to evaluate the association between polymorphisms at D19H, T400K and Y54C and gallstone disease using meta-analysis; 2) to compare the hepatic ABCG5/G8 mRNA expression and biliary lipids composition in patients with different genotypes of T400K and Y54C.

## Methods

### Literature Search

Publication were searched via public database PubMed (http://www.ncbi.nlm.nih. gov/pubmed/), Embase (http://www.embase.com), ISI Wed of Knowledge (http://isiknowledge.com), Wanfang (http://www.wanfangdata.com.cn) and China Biological Medicine (CBM) (http://cbm.imicams.ac.cn) with the last update as of September 2013. The keywords used for search were ‘gallstone disease’ and ‘ATP binding cassette G8 or ABCG8’ combined with ‘gene or variants or polymorphism or alleles’, all of which were MeSH terms (Medical Subject Headings in the US National Library of Medicine). Only studies published in English or Chinese were identified. Afterwards, the full text of the retrieved articles were scrutinized to be sure that data of interest were included. If two or more studies shared the same studied populations, the one with the small size was abandoned. If more than one geographical or ethnic population were included in one paper, each ethnical population was considered separated.

### Inclusion/Exclusion Criteria

Studies that were included satisfied the following criteria: 1. evaluation of the association between ABCG8 polymorphism and gallstone disease; 2. case-control study using either hospital-based or population-based designs; 3) genotype/allele counts of ABCG8 polymorphisms between cases and controls for estimating odds ratio (OR) and 95% confidence interval (95%CI); 4) study population were adults.

Gallstone disease was diagnosed by ultrasonography or operation. In some studies, cholesterol contents in gallstone was analyzed [12], [17]. Studies were excluded if they were published in minor language or published abstracts from meeting.

### Extracted Information

Two authors (ZY Jiang and QC) independently drew the following information from all qualified studies (Table 1): first author’s last name, publication date, population ethnicity, methods to diagnosis of gallstone, study design, methods of genotyping, the distribution of alleles and the genotype in cases and controls. Information such as cases and controls’ age, gender and BMI were also collected.

**Table 1:** List of references.

References	Year	Ethnicity	controls	SNPs	Methods	MAF% of		Number of	
						Cases	Controls	Case	Control
*Xu HL, et al.* (11)	2011	Chinese	Population	D19H/T400K	AD	1.9	1.0	429	443
*Stender S, et al.* (10)	2011	Danish	Population	D19H	AD	13.0	6.1	2894	59385
*Srivastava S, et al.* (12)	2010	Indian	Patients	D19H	PCR-RFLP	5.2	2.5	230	220
*Siddapuram SP,* *et al.* (13)	2010	Indian	Patients	D19H/T400K	ARMS-PCR	8.2	3.8	226	222
*Katsika D,* *et al.* (4) (MZ)	2010	Swedish	Population	D19H	AD	9.1	5.1	44	109
*Katsika D,* *et al.* (4) (DZ)	2010	Swedish	Population	D19H	AD	12.1	5.2	62	126
*Kuo KK, et al.* (14)	2008	Chinese	Population	D19H/Y54C/T400K	AD	4.2	1.0	72	869
*Wang Y, et al.* (17)	2007	Chinese	Patients	D19H/Y54C/T400K	AD/PCR-RFLP	0.87	0.68	287	219
*Grunhage F,* *et al.* (ASP)	2007	Romanian	Patients	D19H/Y54C/T400K	AD	11.9	4.3	84	70
*Buch S, et al.*(15, 19) (I)	2007/2013	Germany	Patients	D19H/Y54C/T400K	AD	11.0	5.6	1266	1000
*Buch S, et al.*(15, 19)(II)	2007/2013	Germany	Patients	D19H/Y54C/T400K	AD	10.7	5.1	1542	1089
*Buch S, et al.*(15, 19)(III)	2007/2013	Chilean	Population	D19H/Y54C/T400K	AD	12.3	7.0	680	442
*Buch S, et al.*(15, 19)(IV)	2013	Danish	Patients	D19H	AD	9.2	5.5	336	766

Abbreviations: AD: allelic discrimination; ARMSR: amplification refractory mutation system; DZ: dizygotic twins; MZ: monozygotic twins; ASP: affected sib pairs; PCR: polymerase chain reaction; RFLP: restricted fragment length polymorphism.

### Patient Recruitment

The study protocol was approved by Ethical Committee at Ruijin Hospital and written informed consent was obtained from each participants. Three panels of patients who subjected to cholecystectomy were included in the analysis. Liver biopsies about 0.5g were taken from the right lobe of liver during the operation and snap-frozen in liquid nitrogen. Part of the samples had been used in our previous studies [20], [21]. Demographic characteristic were described in Table 2. Among these patients, gallbladder bile was collected from 182 patients with gallstone disease and 35 gallstone-free patients (aged: 50.0±0.8 years, BMI: 23.6±0.2 kg/m^2^, female% = 65.0%). All samples were stored in −20°C until laboratory test. Patients with hepatic, renal or endocrine disorders were not included in the study. The gallstones were classified as cholesterol type by cut-face appearance or chemical analysis when necessary as reported [20], [21].

**Table 2:** Demographic characteristic of patients in different panels.

	Panel I (n = 88)	Panel II (n = 100)	Panel III (n = 112)
GS/GSF	59/29	82/19	105/7
Female%	54.5	60.0	59.8
Age	46.8±1.4	48.2±1.2	55.0±1.3
BMI	23.0±0.3	23.5±0.3	24.6±0.3

GS: gallstone group; GSF: gallstone-free control group; BMI: body mass index.

### Gene Expression Analysis

Hepatic total RNA was extracted with Trizol reagent (Invitrogen, Carlsbad, USA) and transcribed into cDNA (ABI cDNA reverse transcript kit, Applied Biosystems, Foster City, CA, USA). Real-time quantitative PCR assays were performed using Sybr-Green (Power Master Mix Sybr Green, Applied Biosystems, Foster City, CA, US). Primers (primer sequences are available on request) were designed using Primer Express 2.0 and all crossing exon-exon boundaries. Data were expressed in arbitrary units, and were normalized by the signals obtained in the same cDNA for Cyclophilin A.

### Genotyping of T400K and Y54C Polymorphism

Single nucleotide polymorphism (SNP) analysis of the polymorphic sites Y54C and T400K in the ABCG8 gene were determined by allelic discrimination using either genomic DNA as template by commercially available Taqman assays (Y54C-rs 4148211: C_29535502_10; and T400K-rs 4148217: C_375061_10, Applied Biosystems, Foster City, CA, USA) or hepatic cDNA as template by Taqman assay developed previously [22].

### Analysis of Biliary Lipids

Biliary cholesterol, total bile acids and phospholipids in gallbladder bile were measured as previously described [23]. Cholesterol saturation index (CSI) was calculated using Carey’s critical table [24].

### Statistics

The associations between genotypes/alleles of Apo E polymorphism with GSD were evaluated by using the software Stata/SE 11.0(StataCorp LP, College Station, USA). In the meta-analysis, we used the random-effects model with the method of DerSimonian & Laird to bring the individual effect-size estimates together. The estimate of heterogeneity was taken from the Mantel-Haenszel model [25]. Heterogeneity was assessed by the *I^2^* statistic, which was documented for the percentage of the observed between-study variability due to heterogeneity rather than chance with the ranges of 0 to 100% [*I^2^* = 0–25%, no heterogeneity; *I^2^* = 25–50%, moderate heterogeneity; *I^2^* = 50–75%, large heterogeneity; *I^2^* = 75–100%, extreme heterogeneity] [26]. Publication bias was evaluated by using funnel plots and the Egger test [27]. The biochemical and gene expression data were expressed as means ± SEM and compared by t-test between genotypes with and without minor allele. Statistical significance level was set as P<0.05.

## Results

### Association of D19H, T400K and Y54C Polymorphisms at ABCG8 with Gallstone Disease: Meta-analysis

**Figure 1:**
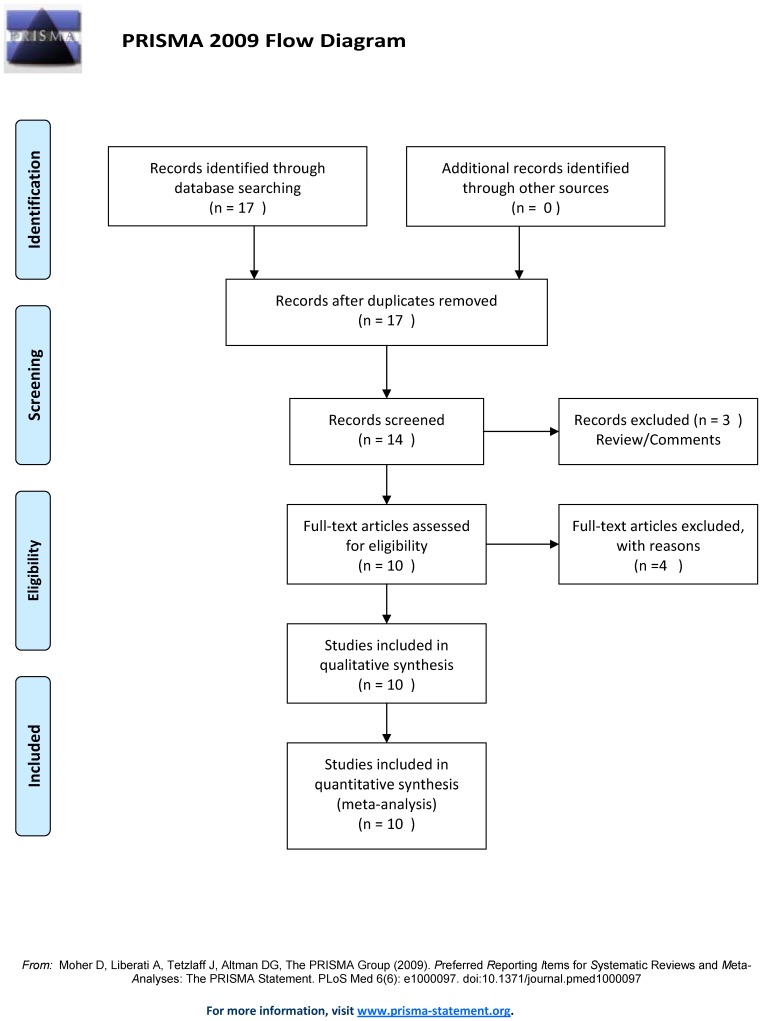
Flow diagram of search strategy and study selection.

We identified 17 papers potentially relevant for our study. Seven papers were excluded and 10 papers including 13 cohorts were included for the final analysis. A diagram schematizing the selection process of identified studies is presented in Figure 1. All studies had genotypic information for D19H polymorphism and only 6 and 4 studies had genotyped polymorphism at T400K and Y54C, respectively. No related meta-analysis was found in the Cochrane-library. Table 1 was presented as the summary of the individuals in this study.

### Genetic Models

**Figure 2:**
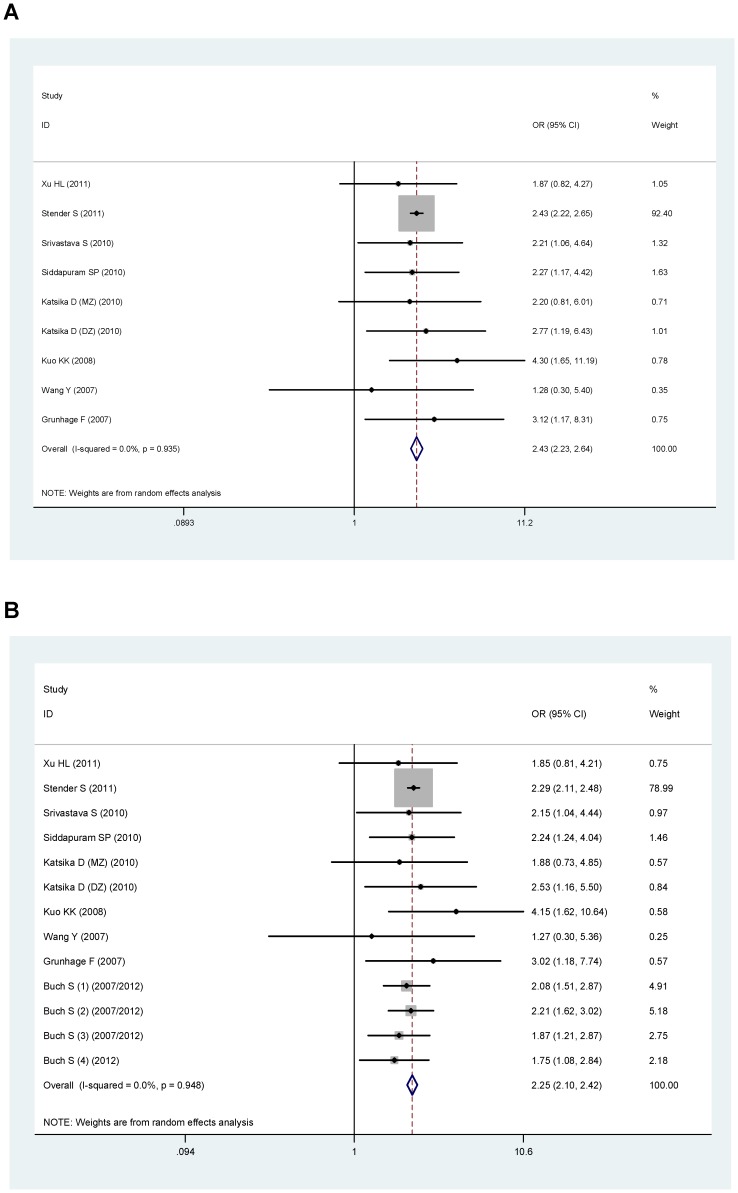
Pooled random-effect-based odds ratio of gallstone disease for D19H polymorphism. A: genotypic model. B: allelic model.

**Figure 3:**
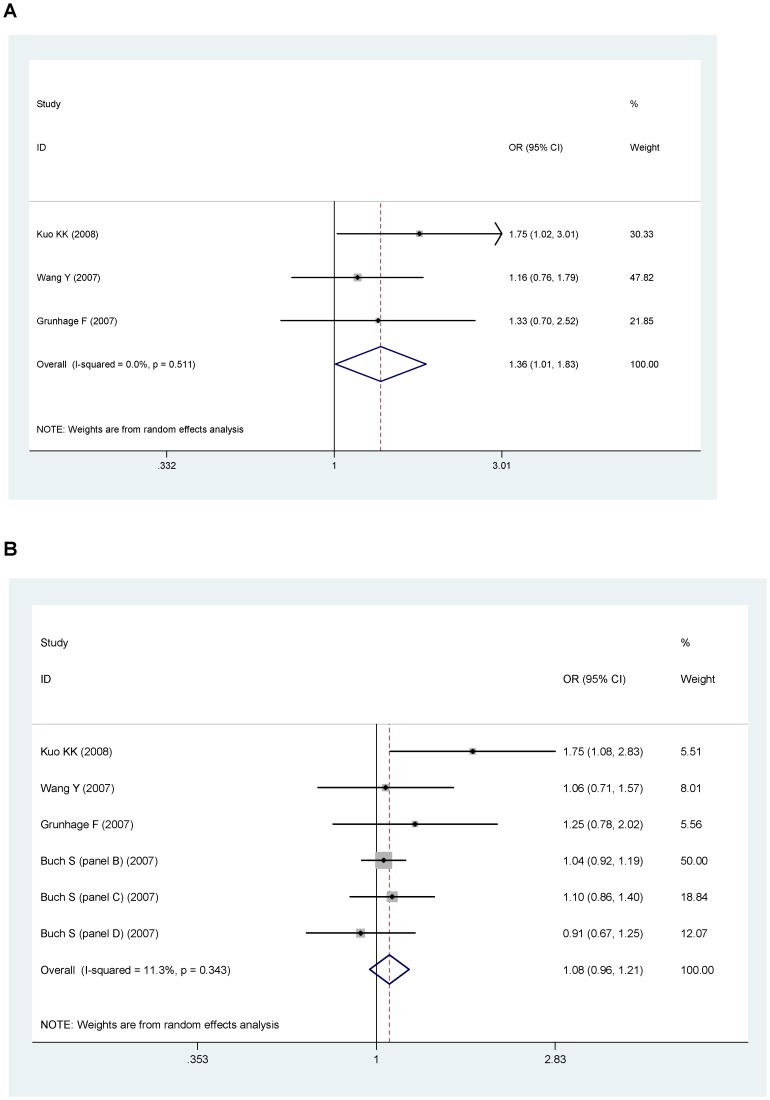
Pooled random-effect-based odds ratio of gallstone disease for Y54C polymorphism. A: genotypic model. B: allelic model.

**Figure 4:**
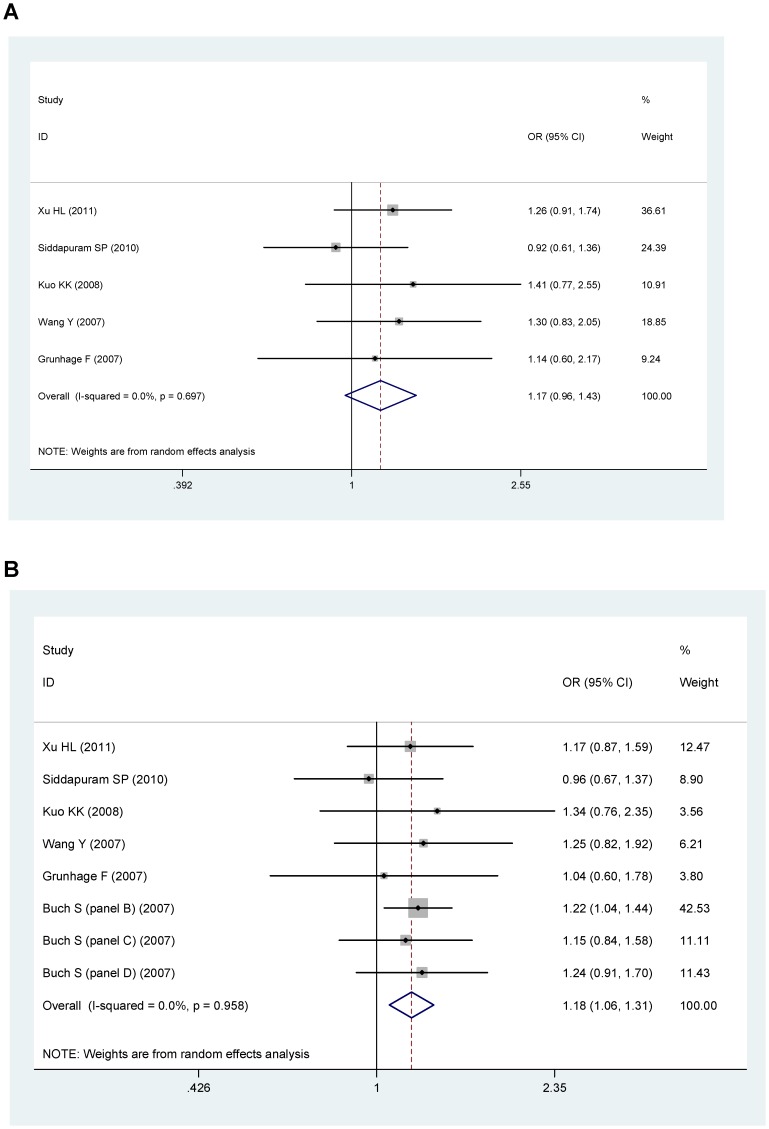
Pooled random-effect-based odds ratio of gallstone disease for T400K polymorphism. A: genotypic model. B: allelic model.

In the genotype model, the overall association between genotype with gallstone was significant for D19H (OR = 2.43, 95%CI: 2.23–2.64, P<0.001, Figure 2A), and for Y54C (OR = 1.36, 95%CI: 1.01–1.83, P = 0.044, Figure 3A), or T400K (OR = 1.17, 95%CI: 0.96–1.43. P = 0.110, Figure 4A).

In allelic model, minor alleles of D19H polymorphism (allele D: OR = 2.25, 95%CI: 2.10–2.42, P<0.001, Figure 2B) and of T400K polymorphism (allele K: OR = 1.18, 95%CI: 1.06–1.31, P<0.001, Figure 4B) were related with an increased risk of gallstone disease. However, minor allele of Y54C polymorphism (allele Y, OR = 1.08, 95%CI: 0.96–1.21, P = 0.146, Figure 3B) was not related with gallstone disease. Because the study by Stender et al., accounted for majority of the overall weight (Figure 2A and B), to eliminate the possible bias, we performed a sensitivity analysis by removing this study in the analysis of D19H polymorphism. However, this did not affect the association between D19H polymorphism and gallstone disease (genotype model: OR = 2.42, 95%CI: 1.78–3.29, P<0.0001; allele model: OR = 2.11, 95%CI: 1.81–2.46, P<0.0001).

### Subgroup Analysis

To compare the difference between different ethnicity, we first divided the studies into Western and Asian population. Significant association between D19H polymorphism and gallstone disease was present both in Western population (allele model: OR = 2.25; 95%CI: 2.09–2.42, P<0.0001, Figure S1A) and in Asian population (allele model: OR = 2.26, 95%CI: 1.58–3.23, P<0.0001, Figure S1A). Therefore, no difference was observed between ethnicities. In the genotype model, the association was also significant independent of ethnicity (Figure S2A). Next, we divided the studies into Chinese and non-Chinese. The association between D19H polymorphism and gallstone disease still existed (Figure S1B and S2B). Due to limited studies for T400K and Y54C polymorphism, comparison between ethnicities was not performed.

Because part of the studies used subjects from general population as controls while the others used patients in hospital but without gallstone disease as controls, we performed a subgroup analysis divided according to the source of controls. As shown in Figure S3, differences in controls did not affect the association between D19H polymorphism and gallstone disease. Furthermore, the sensitivity analysis was performed in the subgroup analysis by removing Stender et al’s study and the above-mentioned association remained (Data not shown).

### Test of Heterogeneity and Publication Bias

*I* ^2^ statistics indicated that no significant between-study heterogeneity for all genetic models for any of the three polymorphisms.

**Figure 5:**
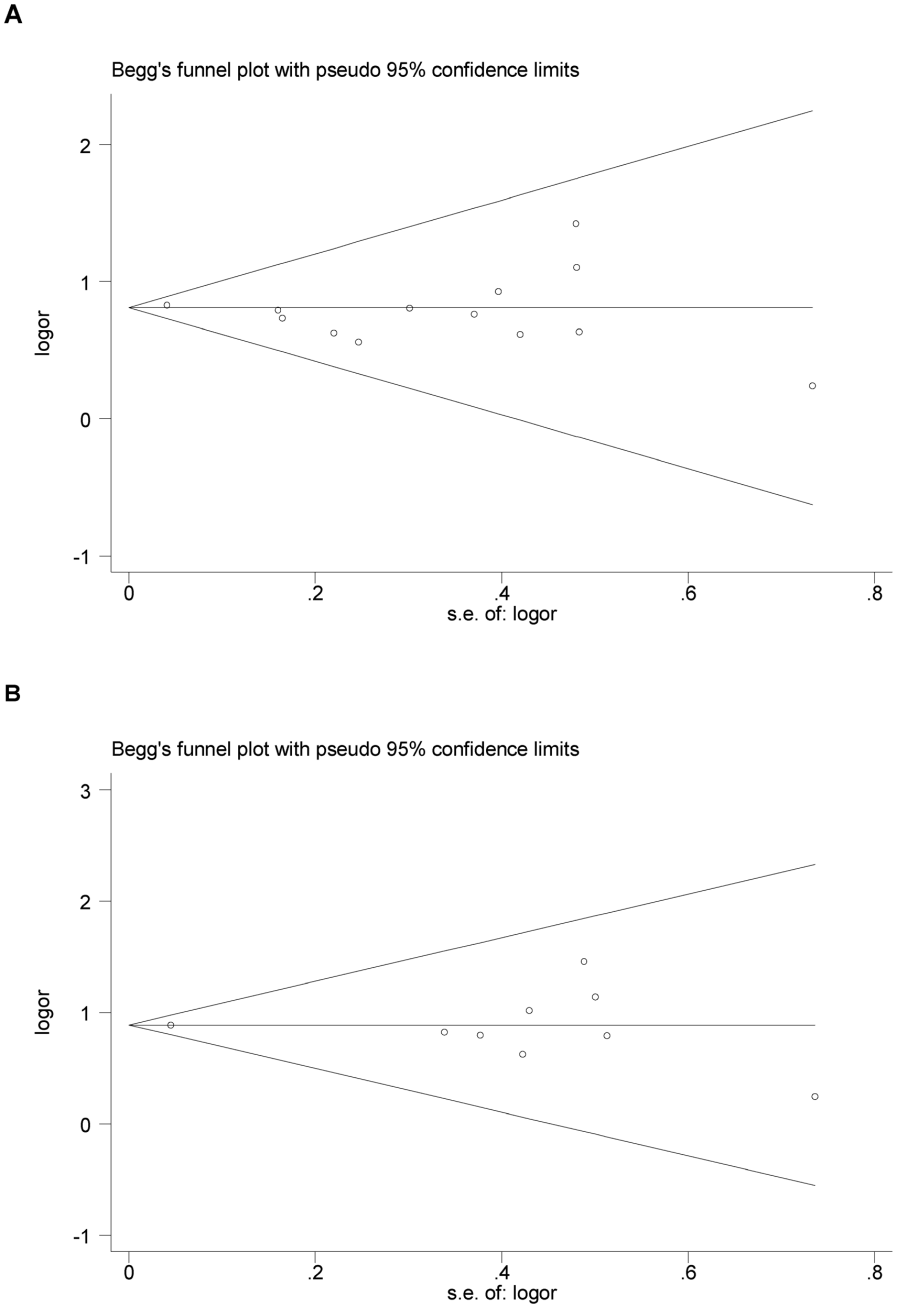
Begg’s funnel plot of the Egger’s test for publication bias of D19H polymorphism and gallstone disease. The horizontal line in the funnel plot indicates the fixed-effects summary estimates, and the sloping lines indicate the expected 95% CI for a given standard error. (A) Allelic model. (B) Genotypic model.

Next, we used Funnel plot and Egger’s test to assess the possibility of publication bias. The resultant symmetrical funnel shape was consistent with an absence of publication bias in the funnel plot for comparison of D19H D allele vs H allele (P_egger’s_ test = 0.400) and DD genotypes vs DH/HH genotypes (P_egger’s_ test = 0.937), Figure 5A and B. No publication bias was present in the funnel plot for comparison of T400K K allele vs T allele (P_egger’s_ test = 0.511), KK/TK genotype vs TT genotype (P_egger’s_ test = 0.815), or Y54C Y allele vs C allele (P_egger’s_ test = 0.274), YY/YC genotype vs CC genotype (P_egger’s_ test = 0.645).

### No Association of T400K and Y54C Polymorphism with Hepatic ABCG8/G5 mRNA Expression or Biliary Lipids Composition

In three panels of patients with liver biopsies, the mRNA expression of ABCG5 and ABCG8 were determined (Table 3). However, the mRNA expression of either ABCG8 or ABCG5 did not differ between the carriers with homozygote of major allele and carriers with minor alleles, except in panel II, ABCG5 expression, unexpectedly, was lower in carriers with homozygote of major alleles than in carriers with minor allele. No difference was observed even after adjusted with age, gender, presence of gallstone and body mass index (data not shown).

**Table 3:** Comparison of gene expression between genotypes (means±SEM).

		T400K			Y54C	
		ABCG5	ABCG8		ABCG5	ABCG8
PANEL I	11 (n = 72)	1.00±0.05	1.00±0.05	11(n = 61)	1.00±0.05	1.00±0.06
	12/22(n = 16)	1.07±0.07	0.95±0.08	12/22(n = 27)	0.94±0.06	0.83±0.06
PANEL II	11 (n = 67)	1.00±0.04	1.00±0.05	11 (n = 66)	1.00±0.04	1.00±0.04
	12 (n = 33)	0.77±0.04**	0.88±0.05	12 (n = 33)	0.78±0.04**	0.88±0.05
PANEL III	11 (n = 83)	1.00±0.04	1.00±0.04	11 (n = 78)	1.00±0.04	1.00±0.04
	12 (n = 28)	1.00±0.08	1.01±0.05	12 (n = 32)	0.99±0.07	0.97±0.05s

‘1’ stands for major allele and ‘2’ stands for minor allele. ** P<0.01 compared to homozygote of major alleles.

Table 4 showed the biliary composition between genotypes. Although, carriers of homozygote of the major alleles of either T400K or Y54C polymorphism tended to have slightly higher biliary cholesterol concentration, cholesterol molar percentage and CSI than carriers of one or two minor allele, the difference was not statistically significantly.

**Table 4:** Comparison of biliary lipids composition between genotypes (means±SEM).

	T400K		Y54C	
	11	12/22	11	12/22
Case	156	57	152	60
Cholesterol (mmol/L)	15.2±0.4	16.7±0.9	15.2±0.4	16.7±0.9
Phospholipids (mmol/L)	46.8±1.4	44.5±1.8	46.7±1.4	44.9±1.9
Bile acids (mmol/L)	158.8±4.6	157.4±6.7	158.7±4.6	157.7±6.6
Cholesterol molar%	7.1±0.2	7.4±0.2	7.1±0.2	7.4±0.2
Phosphopilids molar%	21.4±0.4	20.5±0.4	21.5±0.4	20.4±0.4
Bile acids molar%	71.4±0.5	72.1±0.5	71.4±0.5	72.2±0.5
Total lipids (g/dL)	12.0±0.3	11.9±0.4	12.0±0.3	11.9±0.5
CSI	1.02±0.03	1.06±0.03	1.02±0.02	1.07±0.04

‘1’ stands for major allele and ‘2’ stands for minor allele.

## Discussion

In this study, we collected data from 10 papers which comprised of Danish, Germany, Swede, Romanian, Chilean, Indian and Chinese. Using meta-analysis, we found that carrying H allele of D19H polymorphism was associated with an increased risk of gallstone disease. We also showed that carrying Y allele of Y54C polymorphism and carrying K allele of T400K polymorphism were associated with gallstone disease in either genotype or allele model. However, we could not confirm any difference in hepatic mRNA expression of ABCG5/G8 or biliary lipids composition in association with T400K and Y54C genotypes in Chinese patients.

D19H of ABCG8 gene is the most frequently studied polymorphism in association with gallstone disease. Two studies [10], [15] used large samples. Buch et al [15] showed D19H polymorphism was associated with gallstone disease using GWA approach. Majority of the population they studied was Germany with only a small fraction subjects from Chilean population. The other large sample was all Danish studied by Stender et al [10]. The samples sizes in the rest studies were relatively small [4], [11]–[14], [16], [17]. By pooling all the previous data, 6,328 patients with gallstone and 63,435 gallstone-free subjects were analyzed herein. Our meta-analysis strongly proved the association between this polymorphism and gallstone disease.

An obvious difference of gallstone prevalence between ethnicities is present. Gallstone disease is the highly prevalent in Pima Indians, Hispanic and is relatively lower in Asian and lowest in African population [1]. The frequency of D allele of D19H polymorphism in the gallstone-free subjects is lower in Asian population, from 1% in Chinese [17] to 3.8% in Indian [13]. In Western population, its frequency is between 4.2 [16]–6.9% [15]. Therefore, we divided the population into Asian and Western. Similar results were present between D19H polymorphism in relation with gallstone disease in both populations. Furthermore, if dividing the population into Chinese and non-Chinese, the association of D19H polymorphism with gallstone disease did not change. These results strongly supported that H allele of D19H polymorphism at ABCG8 gene is a common allele predicting susceptibility to gallstone disease independent of ethnicities.

T400K and Y54C polymorphisms were the other two common sites at ABCG8 gene. Not all of the studies retrieved had genotyped either T400K or Y54C polymorphisms of ABCG8 gene. This limited us for a comprehensive analysis of these two polymorphisms with gallstone disease. In the rest 6 studies consisting of 8 populations, we could find that these two loci associated with gallstone disease, at either genotype or allele model. The polymorphic site of a gene can either affect the expression or function of its coding protein of that gene. Unfortunately, we did not find any difference of hepatic ABCG8 and ABCG5 mRNA expression between carriers of different alleles. This suggests that these two alleles *per se* do not affect the ABCG8 expression at least at mRNA level. The coding nucleotide for amino acid is substituted from ACG to AAG and change threonine to lysine for T400K polymorphism, from TAC to TGC and change tyrosine to cysteine for Y54C polymorphism. We could not exclude the possible difference at post-transcriptional and translational level. Furthermore, the function of ABCG5 and ABCG8 proteins as cholesterol transporters depends on the formation heterodimers and translocation from Golgi to apical membrane [28], [29]. The difference might also lie in such processes. Indirect evidence that subjects with KK/TK genotypes had the lower plant sterol concentration suggested an enhanced functionality of the ABCG5/G8 heterodimers [30]. Another possibility is that these two sites may be highly in linkage disequilibrium with another polymorphic site that affects the expression or function of the protein.

We did not observe any difference in cholesterol level in gallbladder bile between carriers of different alleles either. Hepatic bile is usually concentrated by proteins as aquaporin 1 and 8 in the gallbladder [31] and forms gallbladder bile. Other proteins involving cholesterol and bile acids transportation are present in the gallbladder epithelium [32]. Therefore, the possible difference in the secretion rate in hepatocytes between genotypes might be obscured by the resulted biliary cholesterol content due to the presence of various regulatory protein located in the gallbladder epithelium.

Collectively, using a meta-analysis approach, we show strong association of D19H polymorphism with gallstone disease. T400K and Y54C polymorphism, though to a less extent, may also relate with gallstone disease. Further studies on the functional contribution of these polymorphisms to the difference of ABG8 and ABCG5 function in cholesterol transportation will provide more information on their roles to promote gallstone formation.

## Supporting Information

Figure S1 **Subgroup random-effect-based odds ratio of gallstone disease for D19H polymorphism (allelic model).** A: Asian and Western population. B: Chinese and non-Chinese population.
(TIF)

Figure S2 **Subgroup random-effect-based odds ratio of gallstone disease for D19H polymorphism (genotypic model).** A: Asian and Western population. B: Chinese and non-Chinese population.
(TIF)

Figure S3 **Subgroup random-effect-based odds ratio of gallstone disease for D19H polymorphism (population vs patient based controls).** A: Genotypic model. B: Allelic model.
(TIF)

Checklist S1 **Prisma checklist.**
(DOC)
