# Supplementary figures and images for: Association of Three Common Single Nucleotide Polymorphisms of ATP Binding Cassette G8 Gene with Gallstone Disease: A Meta-Analysis
Source: PLoS One. 2014 Jan 30;9(1):e87200. doi: 10.1371/journal.pone.0087200 (PMC3907501; doi:10.1371/journal.pone.0087200)

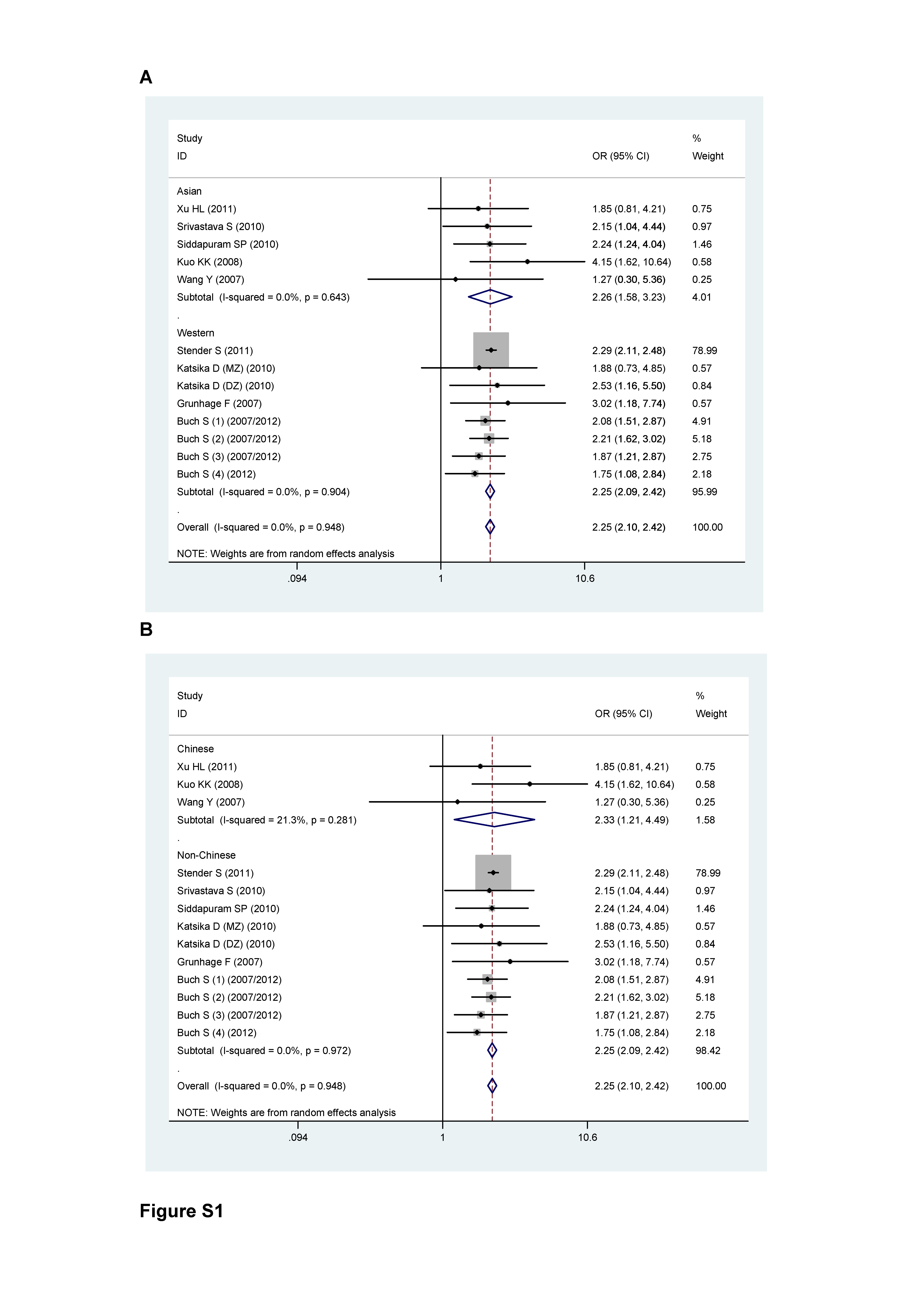

Supplement: Figure S1 — Subgroup random-effect-based odds ratio of gallstone disease for D19H polymorphism (allelic model). A: Asian and Western population. B: Chinese and non-Chinese population. (TIF) [file pone.0087200.s001.tif]

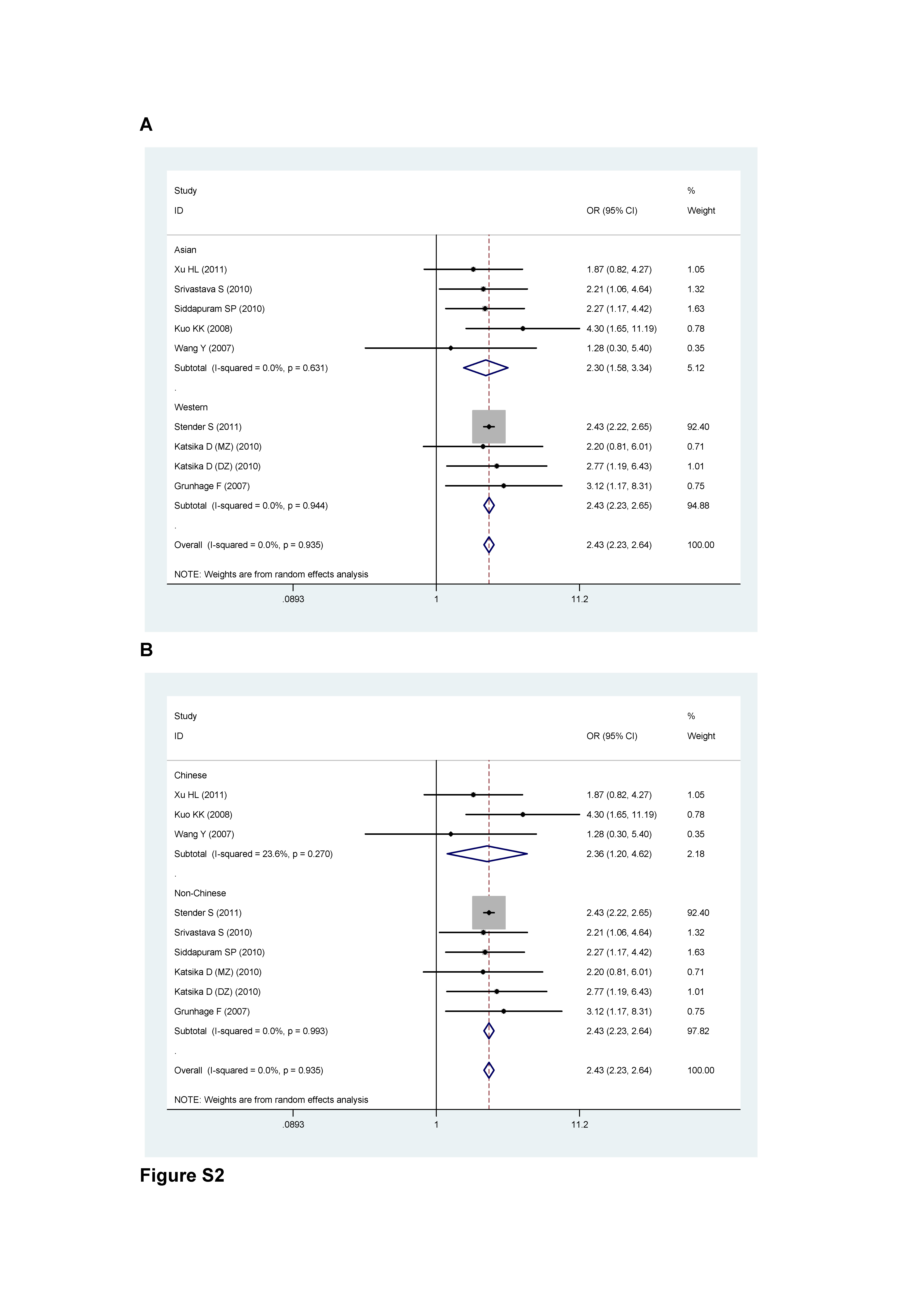

Supplement: Figure S2 — Subgroup random-effect-based odds ratio of gallstone disease for D19H polymorphism (genotypic model). A: Asian and Western population. B: Chinese and non-Chinese population. (TIF) [file pone.0087200.s002.tif]

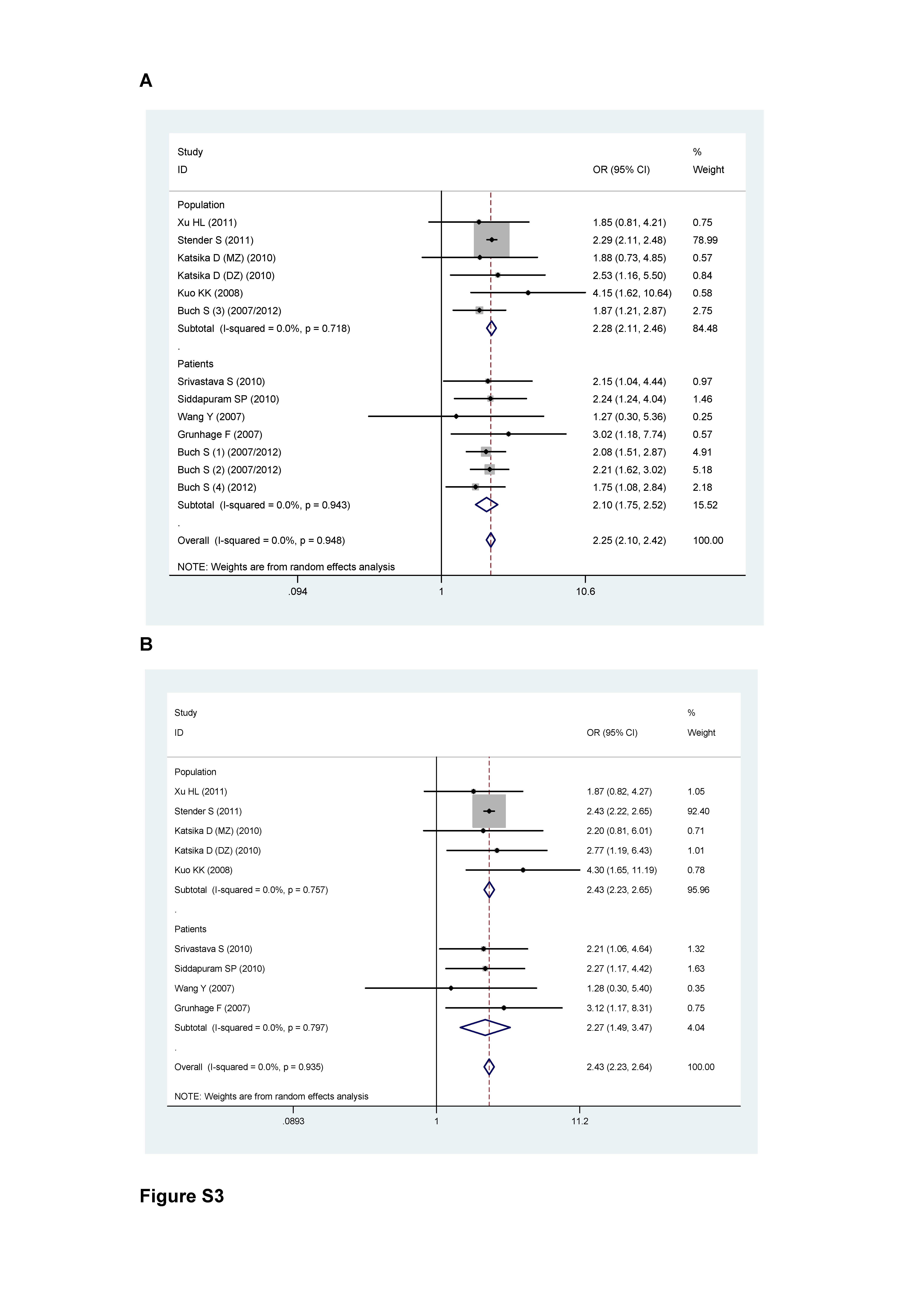

Supplement: Figure S3 — Subgroup random-effect-based odds ratio of gallstone disease for D19H polymorphism (population vs patient based controls). A: Genotypic model. B: Allelic model. (TIF) [file pone.0087200.s003.tif]
